# Supplementary material for: Pneumococcal Extracellular Vesicles Mediate Horizontal Gene Transfer via the Transformation Machinery
Source: bioRxiv. 2023 Dec 15:2023.12.15.571797. Preprint. [Version 1] doi: 10.1101/2023.12.15.571797 (PMC10760141; doi:10.1101/2023.12.15.571797)
Supplement: 1 [file NIHPP2023.12.15.571797v1-supplement-1.pdf]

bioRxiv preprint doi: https://doi.org/10.1101/2023.12.15.571797; this version posted December 15, 2023. The copyright holder for this preprint (which was not certified by peer review) is the author/funder, who has granted bioRxiv a license to display the preprint in perpetuity. It is made available under aCC-BY 4.0 International license.

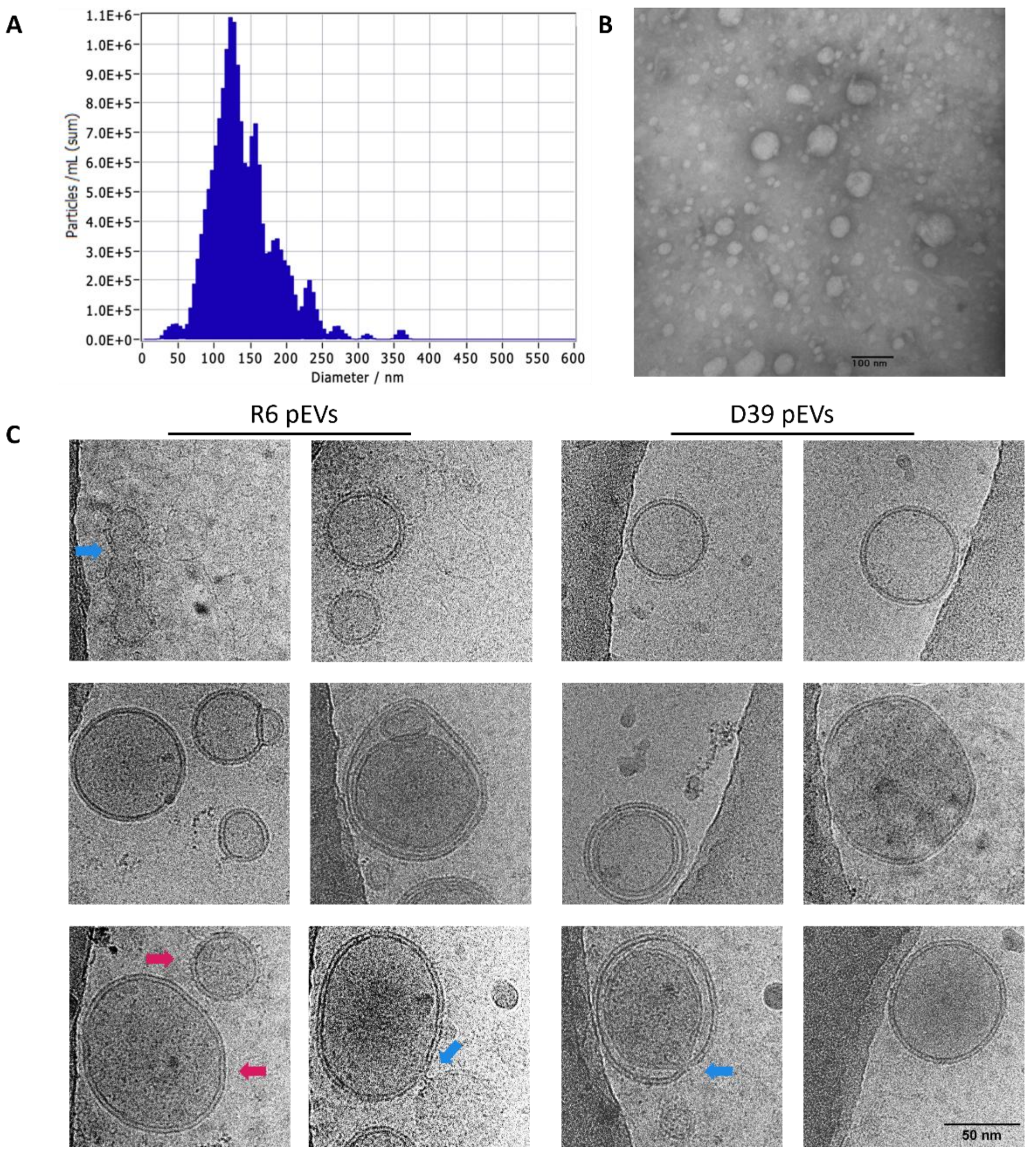

SFig.1: (**A**) Representative nanoparticle tracking analysis of pneumococcal extracellular vesicles isolated from R6 (produced by the software ZetaView). (**B**) Representative transmission electron micrograph of pEVs from R6, scale bar 100 nm. (**C**) Images selected from cryo-electron micrographs of pEVs from R6 and D39. The first row displays representative features observed across most images. Magenta arrows indicate two pEVs representing either smooth or textured surfaces. Blue arrows indicate apparent fusion or fission events (rare in our set). Doublet and triplet pEVs are also relatively rare in our set. All images are the same scale (scale bar of 50nm in final image).

bioRxiv preprint doi: https://doi.org/10.1101/2023.12.15.571797; this version posted December 15, 2023. The copyright holder for this preprint (which was not certified by peer review) is the author/funder, who has granted bioRxiv a license to display the preprint in perpetuity. It is made available under aCC-BY 4.0 International license.

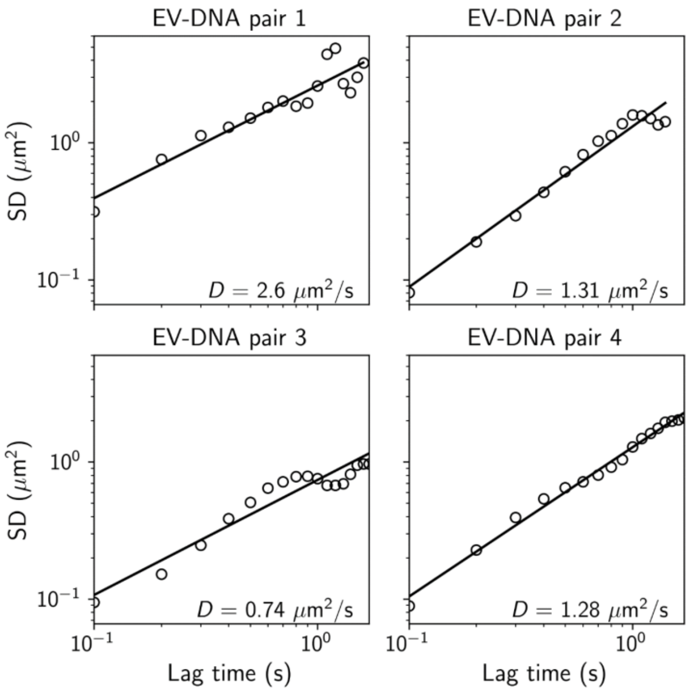

SFig.2: pEV particles follow Brownian trajectories. Points represent computed squared displacements (SD) as a function of lag time for tracked pEV particles. Lines represent linear fits to the data in log-space for each pEV particle. D = Diffusion coefficients.

SVideo.1: Co-localization and co-diffusion of pEV and DNA particles. DNA particles are false colored in green (left panel), pEV particles are false colored in magenta (middle panel), and the merge is present in the right panel. pEV sample was treated with PicoGreen and DiD to label DNA and the pEV membrane, respectively. Scale bar is indicated.

bioRxiv preprint doi: https://doi.org/10.1101/2023.12.15.571797; this version posted December 15, 2023. The copyright holder for this preprint (which was not certified by peer review) is the author/funder, who has granted bioRxiv a license to display the preprint in perpetuity. It is made available under aCC-BY 4.0 International license.

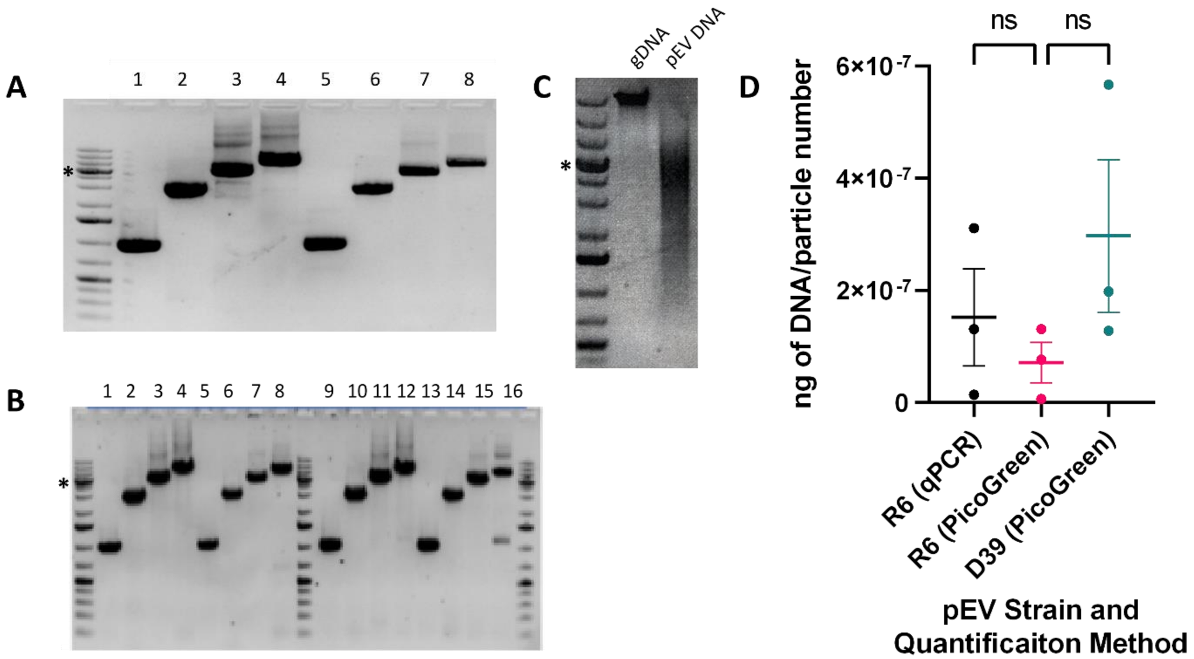

SFig.3: **(A-B)** PCR amplification of 1, 3, 5, and 7 kb DNA fragments from R6 pEVs and genomic DNA. **(A)** PCR primers targeted to gene spr1608. Left lane is the MW standard; lanes labeled 1-4 are positive control where genomic DNA was used as template, and lanes 5-8 used pEVs as template. PCR was performed on two independent sets of vesicles. **(B)** PCR primers targeted to gene spr0001 (left half of the gel) and spr0065 (right half of the gel). Lanes 1-4 and 9-12 are positive control where genomic DNA was used as template, lanes 5-8 and 13-18 used pEVs as template. **(C)** Gel electrophoresis profile of DNA purified from bacterial culture (gDNA) or SEC-purified pEVs (pEV DNA). Asterisk (*) indicates 5,000 base pair marker on the GeneRuler 1 kb Plus DNA Ladder (Invitrogen). **(D)** Quantification of pEV DNA by qPCR and PicoGreen. qPCR was performed on three R6 pEV samples and each point represents four technical replicates. PicoGreen staining was performed on the same three R6 pEV samples and three D39 pEV samples. These data are normalized to the number of pEVs in each sample as measured by NTA. One-way ANOVA compared to R6 (PicoGreen), ns=p>0.5.

bioRxiv preprint doi: https://doi.org/10.1101/2023.12.15.571797; this version posted December 15, 2023. The copyright holder for this preprint (which was not certified by peer review) is the author/funder, who has granted bioRxiv a license to display the preprint in perpetuity. It is made available under aCC-BY 4.0 International license.

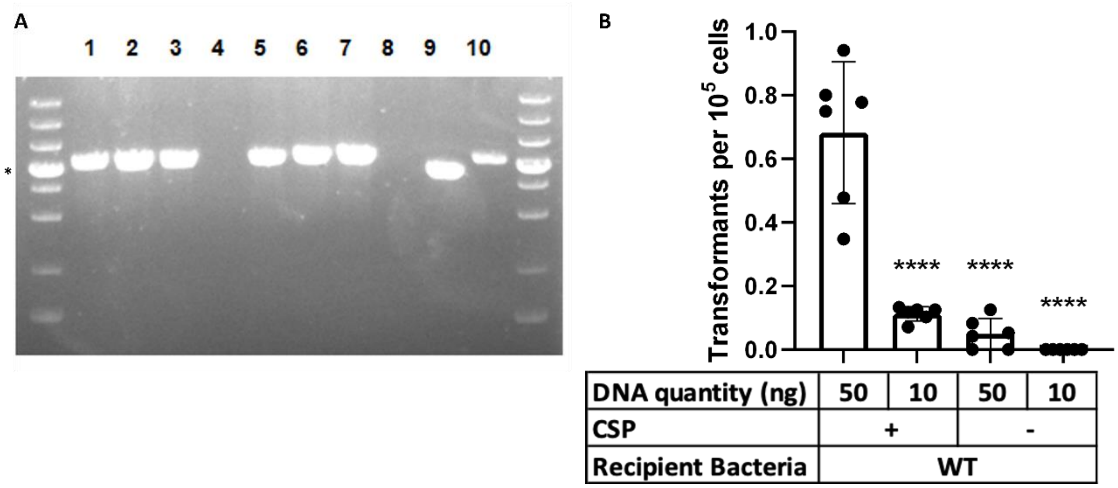

SFig.4: pEVs mediate horizontal gene transfer. **(A)** Three colonies from the transformation plates were grown in rich media overnight and used as a PCR template to check for presence of genes encoding spectinomycin resistance. Every colony produced amplicons of the appropriate size. The templates are as follows: Lanes 1-3 and 5-7: transformation colonies; lane 4: growth media; lane 8 no template; lane 9: gDNA from wild-type R6 SpecS; and lane 10: genomic data from donor bacteria R6-SpecR. Asterisk (*) indicates 5,000 base pair marker on the GeneRuler 1 kb Plus DNA Ladder (Invitrogen). **(B)** D39 cells (SpecS background) were exposed to pEV DNA from a D39-SpecR strain. Transformations were performed with and without CSP. Bars represent mean + SEM with dots overlayed within a bar representing a data point from each independent experiment (n=6, **** adjusted p-value < 0.0001 for Dunnett's multiple comparison test).

bioRxiv preprint doi: https://doi.org/10.1101/2023.12.15.571797; this version posted December 15, 2023. The copyright holder for this preprint (which was not certified by peer review) is the author/funder, who has granted bioRxiv a license to display the preprint in perpetuity. It is made available under aCC-BY 4.0 International license.

Table S1. Primers for pneumococcal regions

Target gene	Primer Name	Sequence
spr.0001	R6.peg.1 F	AGGTAGAGGAAAATGTTGCCA
	R6.peg.1 1026 bp R	CACCATAAAAGTTACCAACTTCAGT
	R6.peg.1 3000 bp R	AATGGGTAGTTTGCTGCCTC
	R6.peg.1 5000 bp R	CCCTTTCTTAGATGAATCAGTCAA
	R6.peg.1 7000 bp R	ATTCCCAGCATAGACATATGGA
spr.0065	R6.peg.65 F	ATGAAAGCATACACAGAGCGTG

bioRxiv preprint doi: https://doi.org/10.1101/2023.12.15.571797; this version posted December 15, 2023. The copyright holder for this preprint (which was not certified by peer review) is the author/funder, who has granted bioRxiv a license to display the preprint in perpetuity. It is made available under aCC-BY 4.0 International license.

	R6.peg.65 1000 bp R	AGGTTTGACCAGCTTTAAGAATG
	R6.peg.65 3000 bp R	AGGTGGTATGAATACTTGGCCC
	R6.peg.65 5000 bp R	GTGAGCTTTCAACTTGGACAAA
	R6.peg.65 7000 bp R	AGCCTTTGAAGAACTTTTACTAGAAGAC
spr.1412	R6.peg.1411 F	GCTCGCTTTGAAGATAGGTTT
	R6.peg.1411 1050 bp R	CCTGCACCAAGAGATTAAAATC
	R6.peg.1411 3053 bp R	TAATCGGGATTCGGATGG
	R6.peg.1411 5029 bp R	TAATCTTATAAGCACCTGCAAGG
	R6.peg.1411 5000 bp R	GCCATCCGAAGATGACTTTT
spr.1825	spGAPDH F	GGTCGTCTTGCTTTCCGTCGTATC
	spGAPDH R	GCTTTCATAGCTGCGTTCACTTCATC
spr.1608	R6.peg.2149 F	ATGAGATACATAACTCTTGGTCAAGATG
	R6.peg.2149 1018 bp R	CCTTTAATGTCAATAATTCTCCCC

bioRxiv preprint doi: https://doi.org/10.1101/2023.12.15.571797; this version posted December 15, 2023. The copyright holder for this preprint (which was not certified by peer review) is the author/funder, who has granted bioRxiv a license to display the preprint in perpetuity. It is made available under aCC-BY 4.0 International license.

	R6.peg.2149 3004 bp R	GTTCGTTTGGTCTAGTTGCAAC
	R6.peg.2149 5000 bp R	AAAATGGCTCTCGAACTGAGTA
	R6.peg.2149 7000 bp R	AATACATGAGCAGGAAGGATAACA

Table S2. Gibson assembly primers and sequence check primers

Construct	Primer Name	Sequence
1900 Spec	14381 F1_fwd	CGTGAAGCTATCCAAGAAGAAGC
	14382 F1_rev	ATATATGGATCCCTGCTCCTAGGGAGATTTATCTTT
	14383 F2_fwd	ATATATCCCGGGGTCGCTCCGTCGTTCGATTTCCTA
	14384 F2_rev	CCATCTCAGAGATATGAACAAGGGCATCTGTC
	14385 1900specCassette_fwd	TTACCGTAAAGGTGAATTGT
	14386 1900specCassette_rev	TAAACTTCATCCACTTTGGC
	13021 Spec_fwd	ATATGGATCCTCCCCCGTTTGATTTTTAATGGTAA TGTGATAAA
	13022 Spec_rev	ATATCCCGGG CGGAATGGATCCAATTTTTTTA
ΔcomEA/comEC	comEA/EC KO F1_fwd	GGTACACCACCAAGTAGAAC
	comEA/EC KO F1_rev	TAGGCATAGACTCGATAATTGCTTCCATATTTTC
	comEA/EC KO Kan Term_fwd	AATTATCGAGTCTATGCCTATTCCAGAGGAAATGG
	comEA/EC KO Kan term_rev	TCGATTTTCCGCGAAAAAACCCCGCCGAAG
	comEA/EC KO F2_fwd	GTTTTTTCGCGGAAAATCGAAAGT GTTCG
	comEA/EC KO F2_rev	CCAAGCTGACTGAGTTTG
1900 Spec	Spec_seq_Up	GGA TGA TTC CAC GGT ACC
1900 Spec	Spec_seq_Down	GGG AGA GAA TTT TGT TAG CAG TT
ΔcomEA/comEC	Kan_seq_Up	CCACCAGCTTATATACCTTAGC
ΔcomEA/comEC	Kan_seq_Down	GGACAAGTGGTATGACATTGC
